# Ten step academic-industry digital health collaboration methodology: A case-based guide for digital health research teams with the example of cardio-oncology

**DOI:** 10.3389/fcvm.2022.982059

**Published:** 2022-09-30

**Authors:** James MacLeod, Mohamed Abdelrahim, Sabrina Painter, Ragasnehith Maddula, Austin Steward, Abdulaziz Hamid, Richard K. Cheng, Vlad Zaha, Daniel Addison, Brenton Bauer, Sherry-Ann Brown

**Affiliations:** ^1^Medical College of Wisconsin, Milwaukee, WI, United States; ^2^Joseph J. Zilber School of Public Health, University of Wisconsin-Milwaukee, Milwaukee, WI, United States; ^3^Division of Cardiovascular Medicine, University of Washington, Seattle, WA, United States; ^4^Cardiology Division, University of Texas Southwestern, Dallas, TX, United States; ^5^Cardio-Oncology Program, Division of Cardiovascular Medicine, The Ohio State University, Columbus, OH, United States; ^6^COR Healthcare Associates, Torrance Memorial Medical Center, Torrance, CA, United States; ^7^Cardio-Oncology Program, Division of Cardiovascular Medicine, Medical College of Wisconsin, Milwaukee, WI, United States

**Keywords:** digital health (eHealth), academic-industry collaboration, cardio-oncology, digital transformation, research guide, industry-institute collaboration

## Introduction

Novel technologies developed by industry are increasingly being applied in healthcare (1). Established corporations as well as start-ups are rapidly developing digital health products to leverage the multitude of sensors, trackers and digital tools contained within smartphones, laptops, and other technologies. With these products and the data they collect come promising routes of application in various medical fields. One discipline that could benefit from management of patient risk factors through digital health implementation is cardio-oncology, a relatively new specialty focused on the optimization of cardiovascular care for individuals with cancer (2, 3). This manuscript explores the creation of an academic-industry collaboration to help fill the unmet need of managing cardio-oncology patient risk factors through digital health and provides ten case-based steps that can be replicated by others seeking to conduct similar research.

**Figure 1:**
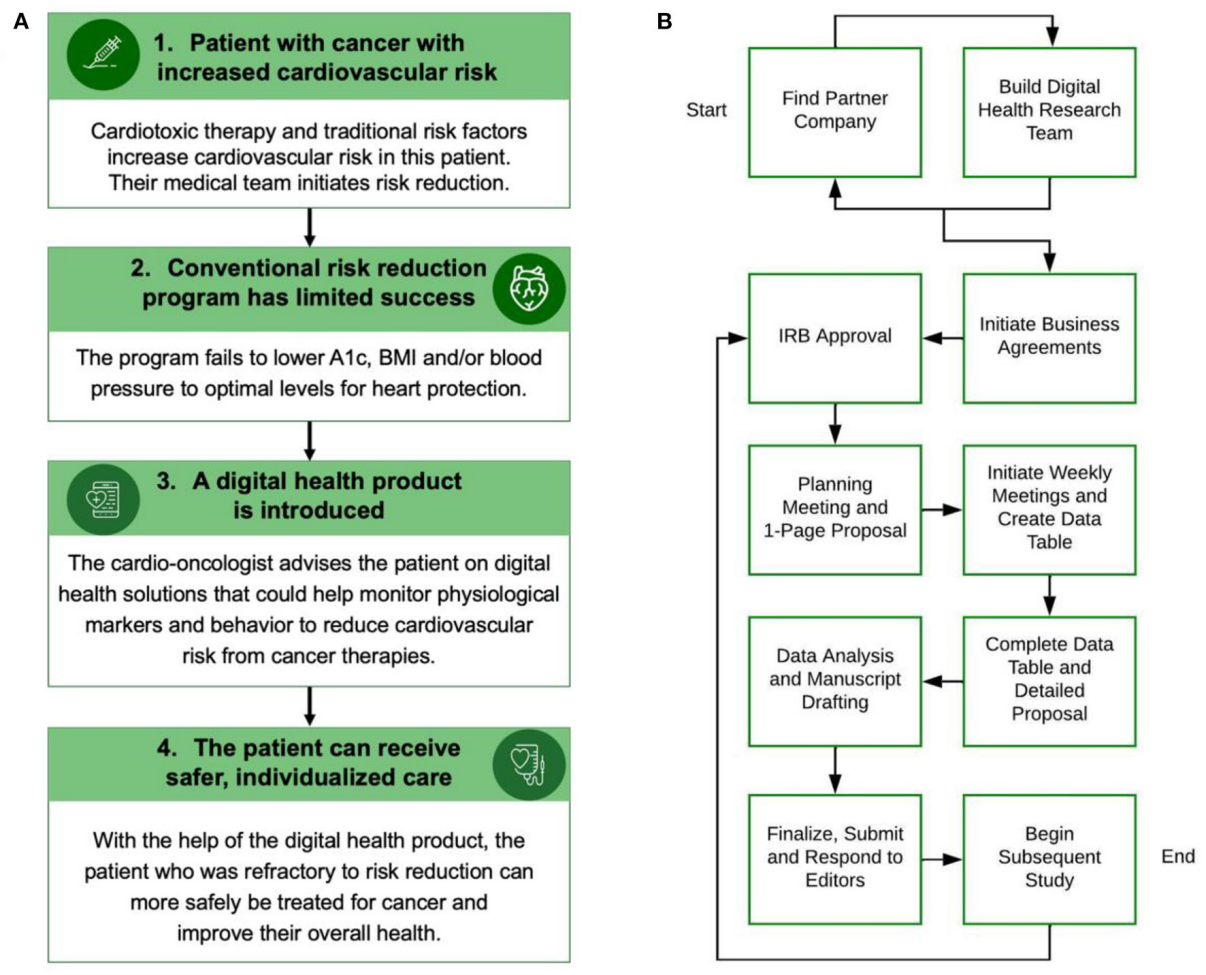
**(A)** This figure shows how a cardio-oncologist could integrate a digital health product into risk reduction for a cancer patient. BMI: Body mass index. **(B)** This flow chart describes ten steps for building a digital health academic-industry collaboration. The process can begin with finding a partner company, building a digital health research team, or both concurrently. IRB: Institutional Review Board. Panel A was made with Infographia.

Many methods are used in digital health products to monitor user physiology and manage health. These technologies include accelerometry, global positioning systems, electrocardiography, photoplethysmography, oscillometry, barometry, skin conductance, electromyography, video, chemical sensors and sound/microphones (4). As these health-focused products are developed, academia must keep pace to assess safety and efficacy (5). Like many fields of medicine, the cardio-oncology patient population could benefit from safe and effective digital health solutions. Patient-related risk factors such as diet and weight can increase cardiotoxicity risk; managing these risk factors is crucial to improving cancer treatment outcomes and an area where improvement is needed (6, 7). In a 2021 study, women with breast cancer receiving potentially cardiotoxic chemotherapy were found to not show significant improvement in hemoglobin A1c or body mass index while following a comprehensive cardiovascular risk reduction program (8). This study demonstrates that current methods are not enough to reduce these risk factors in some cardio-oncology patients. The use of digital health products in cardio-oncology is a relatively unexplored space that could help address this and supplement current standard of care diet and weight interventions (Figure 1A) (3, 9, 10).

The World Health Organization (11), the Agency for Healthcare Research and Quality (12) and the Food and Drug Administration (13) have comprehensive guides for monitoring and evaluating digital health technologies. However, there is a lack of literature on patient experience and effects on clinical outcomes for specific digital health products (14). Building this body of knowledge is critical to promote patient safety, efficacy and clinical implementation of digital health products (10).

This case-based article explores components of forming a digital health academic-industry collaboration and is applicable to many fields of medicine. Ten steps that were followed to form a relationship with a digital health start-up are provided, followed by a discussion of each step in the context of current literature. This article is meant to help advance partnerships and collaborations, ensure equity in digital health research and educate students at all stages of training. It may also be useful for academicians planning digital health research, digital health companies with an interest in academic collaboration, clinicians looking to integrate digital health into practice, patients using these technologies, and other partners. Hopefully, the work will propel trainees and practitioners into academic-industry collaboration in the digital era.

## Case example: Ten steps describing an academic-industry collaboration

### Identify partner company

The initial connection with the case company occurred by serendipity on a flight returning from an academic society conference. The industry professional was a passenger next to the principal investigator. Their discussion evolved to focus on the digital health start-up in need of clinical and scientific advisors. Through this connection, the possibility of participation in a research relationship was proposed. The company provides complementary services on the continuous glucose monitoring (CGM) software platform, which is coupled with CGM hardware from other independent companies. The CGM hardware uses an intramuscular sensor to transmit real-time blood glucose levels to the user through a smartphone app. Services included in the application are visualized glucose tracking, access to an assigned registered dietitian, and activity, meal and exercise tracking, among other options. At the core of the company's goals is helping users associate daily behaviors with the associated effect on their glucose levels. The company gleans a wealth of data including the forementioned datapoints, as well as questionnaire responses from users at the onset of signing up for the program, in addition to questionnaires after 1 month and also upon completion of using the software system/smartphone app. These tools are a great fit for potentially helping cardio-oncology patients, given that personalized nutritional counseling has been shown to be beneficial for cardiovascular health in general (15, 16) and individuals with cancer (3, 17, 18).

### Build digital health research team

Such interactions with industry helped guide the creation of a research team to suit the needs of the potential academic-industry collaboration. Medical students, graduate students, residents, fellows and attending physicians were recruited through several methods to form a core research team to plan and conduct studies with this start-up and others. Building a research team can also occur before relationships with digital health companies are formed, or concurrently. This particular relationship dwells within the Connected Health Innovation Research program (C.H.I.R.P.), which is part of the broader Cardio-Oncology Innovation Network (COIN) consisting of professionals in cardiology, oncology, and related medical, technological, or regulatory fields (19). The group utilizes quarterly meetings with the broader COIN membership to improve study and program design, by incorporating suggestions from other professionals within the network that have unique perspectives and expertise. Trainee research team members received stipends where possible through departmental student research grants, while physician team members were not directly compensated. On the industry side, the start-up company allocated internal resources to execute statistical analysis and help answer questions regarding company offerings and subsequent data.

### Initiate business agreements

Before commencing formal discussions of detailed research plans, a non-disclosure agreement (NDA) was completed with guidance from the academic institution's general counsel and research provost. This document was completed based on an institution template and signed by the start-up co-founder and the academic institution's research provost. Responsibility for business agreements was shared between the partner company and institution. The NDA outlined intellectual property rights and data ownership including the company maintaining data ownership throughout the process, and with published products remaining the intellectual property of the research organization/publishing journal and not controlled by the industry partner. Collaborating companies such as these would share in the process of data analysis and would not have control over what our team could publish per the business agreements. Privacy of users was ensured through following internal institutional review board (IRB) policies. Potential conflict of interest was also discussed including the C.H.I.R.P. leading physician being a clinical advisor to the company, but not receiving compensation or benefits. Months later, as discussions evolved, and a long-lasting research partnership was agreed upon, a memorandum of understanding was also proposed to ensure that company resources allocated to the partnership would remain fruitful.

### Institutional review board approval

Research team members drafted and completed the retrospective IRB protocol based on an institutional template, under the guidance of the research team's leading physician scientist. The protocol, which was submitted internally, covered similar C.H.I.R.P. projects with several companies. It was noted that these retrospective studies would have no control over confounding variables and thus would require analyzing data from more users than protective studies designed with controls. The collective retrospective project was considered low risk and informed consent was deemed unnecessary due to the de-identified nature of the data. The protocol was accepted 2 months following initial submission. Amendments were then submitted underway to also include prospective studies, such as an upcoming 24-month study collaborating with the CGM start-up and an established company that produces CGM glucose trackers. The CGM start-up company purchases glucose tracking hardware to transmit live glucose data to the user-facing app.

### Planning meeting and one page proposal

In October of 2020, following an email invitation, the chief operating officer of the CGM company gave a live virtual demonstration of the company platform, use cases, and client base, during a weekly Brown Lab research meeting. Prior to this presentation, a one-page proposal document (see template document in the C.H.I.R.P. Compendium) was initiated by the research team shared *via* Microsoft SharePoint. C.H.I.R.P. and the overreaching goals, along with a brief description of the CGM start-up and its use population, were described. This initial document also outlined the general research questions to be answered through the partnership, objectives of the study, and plans for statistical analysis (in a later step we outline how this document was expanded over several weekly meetings with the company). Goals for the initial retrospective manuscript were laid out, to include assessing measures of adoption and clinical outcomes with subsequent publication. A general outline of the project stakeholders was delineated, including on the company side (start-up founders, investors, and users), and on the research program side (academic institution, researchers, clinicians, and future patient users).

### Initiate weekly meetings and create data table

During the planning meeting, the Brown Lab lead physician suggested weekly, 1 h virtual meetings between C.H.I.R.P. and start-up team members. To date, at least 12 meetings have occurred. In the initial weekly meeting, the company was represented by a senior operations manager, medical student intern, data analyst, senior engineer and the chief operating officer. Lab representatives were the C.H.I.R.P. medical student assigned to the company, the lead attending physician and our academic institution statistician. The meeting revolved around identifying the company and research team partnership goals, skillsets and resources. The company emphasized assessing the connection between use of its product and improved health outcomes. The research team sought access to the wealth of user data to assess this same connection and future projects. To achieve these goals, collectively we created a table listing all available types of data parameters, so that useful datasets for the study could be identified and planned (the table template used for this purpose can be found in the C.H.I.R.P. Compendium). Datasets were formed from various in-application user questionnaires, clinical tracking metrics, nutritionist interaction and user feedback surveys.

### Complete data table and detailed proposal

Over the next four weekly meetings we discussed the category, frequency, rigor of abstracting, rigor of analysis, type of data (categorical vs. continuous) and type of data presentation for each company dataset and included this information in the table. The proposal document was cross-referenced to the table and expanded to outline the specific primary and secondary end points based on what was most measurable and what start-up datasets would be analyzed for each one. The proposal document was now over three pages long and thoroughly described all end-points. Through these weeks of discussion, we also covered demographics and limitations of the initial retrospective study including possible lack of diversity in the user population due to cost barriers and lack of quantity or usability for some datasets. Limitations were identified for future incorporation into the study manuscript and to help plan the future prospective study.

During the process of expanding the proposal and data table, expertise and availability at the company and our academic institution for data analysis were discussed. Two physicians with retrospective industry academic study experience were invited to one of the weekly meetings and confirmed the plausibility of having the start-up conduct statistical analysis through their own data team. In addition, supplementary data analysis was to be performed by our academic institution department of medicine statistician. Details pertaining to funding of the study, intellectual property rights, and data ownership were discussed. This study did not have internal or external funding. However, there was little to no expected cost based on the retrospective nature, predominantly student research team.

### Data analysis and manuscript drafting

This was the first academic-industry collaboration for this start-up and they expanded their company to meet the needs of conducting research. The start-up company hired a data team and made the retrospective and prospective studies with C.H.I.R.P. the primary project for one of the hires. C.H.I.R.P. met with our academic institution department of medicine statistician to help with data interpretation on the research team side. The retrospective CGM data needed to be cleaned to ensure all recorded readings were accurate and useable; this process is thoroughly described in the literature (20). The start-up also uses Amazon Web Services cloud storage which complicated CGM data analysis for research purposes. This form of data storage dissuaded one of our leading statisticians from wanting to work on the project given the difficulty in working with this cloud based uncleaned CGM dataset. Ultimately, it took several months to prepare the clean data for analysis. As this process occurred, the manuscript template was created, non-data reliant sections of the manuscript were drafted by C.H.I.R.P. team members, and the future prospective study was planned.

### Finalize, submit, and respond to editors

Upon completion of a manuscript draft, the Brown Lab principal investigator gives broad initial feedback. Additional editors are recommended within the collaborative network. Once editor feedback has been incorporated, the principal investigator comprehensively reviews the manuscript making changes and leaving comments for the student team. After these steps are complete, the manuscript is cross referenced to the journal author guide to be finalized for submission. The research team works together to address post submission editor comments based on who completed the portion requiring changes and availability. It may be difficult for some journals to find reviewers for such manuscripts, due to the scarcity of similar studies in these emerging fields. C.H.I.R.P. considers journals with a focus on collaboration with industry or digital health, or academic reviewers familiar with these areas. Company personnel and resources are often preferred especially for retrospective analysis given the nature of the data collection not being optimized for research purposes and preferably needing to be de-identified before being sent to the academic institution. Reviewers of manuscripts in this area will likely request as much detail as possible about the statistical analysis portion of the study in particular. It is important for the protection of the journal, reviewers, authors, company, and users to ensure analysis was conducted in a way that assures the integrity of results and findings. If the manuscript is not accepted, a plan of action is created at the following weekly lab meeting. Potential solutions include reorienting the manuscript, or finding a more appropriate journal for publication.

### Begin subsequent study

While working on the initial retrospective study, the opportunity for a prospective study involving the same CGM start-up and a second CGM supplying company arose. The research team concurrently began the steps for a subsequent study, while continuing the initial one. The prospective study required a new IRB protocol amendment and study proposal which were planned during the weekly meeting with the start-up, simultaneous with retrospective data planning and analysis. In deciding on how to conduct a subsequent study, we considered how the start-up collects data and whether this would be easily amenable to a prospective format. Importantly, we saw the opportunity to use the mobile application to gain participant consent, and the opportunity to orient data collection to a more research friendly process than their retrospective data. A strong relationship was built with the start-up over several months of communication and continuation of the partnership was mutually beneficial. The company demonstrated a willingness to adapt to participating in research and we had formed a foundation for future work together.

## Discussion

This ten step case-based guide has been established for use by research teams, companies and students interested in creating collaborative relationships with digital health or other industry companies and provides context for existing academic-industry collaboration blueprints. Prior groups have described how to initiate and evaluate an academic-industry collaboration (11, 13, 14, 21), but there is a lack of case examples of these partnerships in the real world (14). The ten steps C.H.I.R.P. followed in forming a partnership with the use case CGM start-up described herein are included to address this literature gap and prompt research programs to conduct similar research (Figure 1B).

The mHealth Impact Lab at the University of Colorado similarly outlined steps taken with four industry technology partners in a case report (22). The same University of Colorado team also assessed benefits of collaboration and recommendations in a separate review manuscript that include a guiding checklist, but not illustrative examples of each item from case-based experience (14). The current guide presented here builds on this case report and review by including case-based specifics for each recommended step. Independently we established ten steps in our research group, which in comparison adds three steps to the seven from the University of Colorado. The three additional steps in our report are 1) the identification of a key partner company step, 2) expansion of the referenced “protocol development” step to include completion of the two C.H.I.R.P. Compendium documents, and 3) a new step delineating data analysis. These steps add value in several ways. Including actionable advice on how to reach out to companies for collaboration, rather than solely companies approaching the institutional team, makes this guide applicable to new research teams most in need of guidance. Providing guiding templates and an explicit description of how they were completed during the collaborative process makes the process easier for other research teams to replicate and build upon. Finally, by giving further detail on data analysis challenges research teams can be more prepared for discussing this topic with companies and planning projects. In addition to providing the ten steps that C.H.I.R.P. has followed with a CGM start-up, we also have developed the C.H.I.R.P. Compendium, which provides templates for the foundational academic-industry collaboration proposal document and data tables.

Digital health academic-industry collaboration can be initiated by interested companies or research programs. This step may occur prior to forming the research team, concurrently or afterwards. Finding a partner company can occur in a multitude of ways including: use of social media including LinkedIn, conferences, online research and society databases and fora, and network connections. In most cases, academic research teams are seeking data collected through the company platform, and companies are seeking academic research supporting their product(s). A digital health research team forms the foundation for digital health technology assessment. Ideally this team should be part of a broader collaborative network where projects can be shared, expedited and receive feedback (19, 21). The particular compilation of team constituents should be customized to reflect the research niche and goals. It will be important, however, that the core team is capable of designing research studies, writing manuscripts and directing data analysis, with some of the team members having such experience prior to joining the team.

Leading into discussions, one to several business agreements between the research institution and the company should be completed. These documents seek to lower risk for stakeholders beginning with an NDA to protect classified company information and the integrity of the research studies, and can also include a data use agreement, business associate agreement and/or memorandum of understanding. The documents should be completed with direct guidance from the research institution's general council. It is made clear during introductory meetings and in business documents that the digital health company does not have control over what is published by the research team. The company gives crucial input on the types of business documents to be executed, to ensure protection of their information and interests in the case of a retrospective de-identified study using data from company software or device adopters. For a prospective study, enrolling patients at a research institution requires more detailed and specific agreements. These documents are crucial to the development of a productive working relationship, given the fundamental differences in academic and industry goals (14). Dynamics will differ when working with a start-up vs. an established larger industrial corporation. For example, a start-up may not have a data analysis and research focused team while an established corporation is likely to have more experience partnering with academic institutions and/or conducting internal research. Alternatively, there may be more options for established companies in terms of selecting partnerships leading them to partner with larger, more experienced research programs.

Regardless of the size of the company, an internal IRB proposal must be created, and can encompass all digital health related research projects of the digital health research group, as in our case. It is suggested to start with a de-identified retrospective study proposal, which is simpler to execute, can be approved more quickly, and will be a smoother mechanism for onboarding of the initial research study team beginning this work. This proposal should be completed in accordance with individual institutional guidelines and is necessary to further discussions with companies. All team members involved in any of the digital health related projects must be included on the protocol. The initial protocol should mention the future addition of a prospective study. Beginning with a retrospective study, where possible, prepares parties for complications and adjustments for future studies. A faster timeline can occur, which the pace of technology advancement demands (5). In our case, retrospective CGM analysis has been shown to be valuable, but in some cases retrospective analysis may not be feasible (20, 23). Companies interested in collaboration are invited to attend weekly lab meetings, to demonstrate their product(s) to lab members. Beginning at the first meeting, a company profile and one-page study proposal document is initiated; a proposal guide is included at C.H.I.R.P. Compendium. The guide provides a brief description of the research program and overarching goals, the company name and offerings, research questions, and study objectives. Industry university collaboration should be mutually beneficial and it is important to openly communicate goals for the collaboration early in the project to develop trust through openness (24, 25). It is expanded to include specific research questions and corresponding data for the study and the study plan in the next step. Lab members interested in working with the presenting company are given leading roles for the communication between the research team and the company, and progressing the timeline to data acquisition, analysis, and publication. It is important to meet frequently especially during initial phases of the relationship to work through challenges as they arise (14).

Following completion of initial planning steps, weekly meetings should be attended by the company team members responsible for research collaborations, data analysis and product engineering. The research team members should include the research team student responsible for the company as well as the leading physician associated with project. In meetings, the study proposal should evolve to include specific research questions, corresponding data, and the study plan including primary endpoint (e.g., CGM data in relation to application use frequency) and secondary endpoints (e.g., change in weight in relation to application use frequency). A table should be made that includes variables classified based on category of research question (e.g., clinical outcomes, adoption), frequency over 6 months (baseline value vs. number of times updated over 6 months), rigor of abstracting data (based on collection methods and type of data), rigor of analysis, type of data (quantitative, qualitative, or categorical) and type of data presentation/analysis (such as amenable to calculating *P*-value, Pearson correlation, time series, and so on). The number of charts and types of associations should be included in the same or a second sheet; an outline of such a table is included at C.H.I.R.P. Compendium.

It is important to discuss limitations of the study in terms of accurately representing the population, as many digital health company offerings have inherent cost barriers to use. Special attention should be given to assessing and including racial and ethnic demographic information of users. This key information is often missing entirely from studies and is crucial to ensuring equitable application of digital health technologies (26, 27). Any situations where this data is unavailable or a user population is not representative of the general population should be noted as a limitation and improved upon for prospective arms of future studies with the company.

Data analysis and manuscript drafting can commence at any time, but after completion of the study proposal and table(s) is ideal. It is important to determine who will complete data analysis, and whether this will be pursued by statisticians within the research team's academic institution or consulting third-party statisticians financed by the company. Research team members should be assigned tasks for manuscript drafting, and progress should be assessed at continued weekly lab meetings. Authorship is assigned based on the distribution of roles in and contributions to each individual manuscript. A general order of authors would be the research team member assigned primary company lead, other research team members who contributed to the manuscript, any statisticians involved, and principal investigator. Upon completion, the manuscript should be reviewed by all authors, field experts within the research team's collaborative network, and the company if applicable without introducing substantial conflict of interest. The final approval before submission should be given by the principal investigator, and should be clarified early on that the company does not guide or restrict publication of the work. The manuscript should be submitted by the corresponding author. The formal peer review process by the journal would then begin and would ideally lead to reviewer comments associated with an invitation for manuscript revision. The research team member assigned to the company and corresponding author should coordinate changes and responses to editor comments. Journals are targeted that have a history of publishing similar studies, or editors familiar with the research team's projects.

This completes the 10 steps for academic-industry collaboration and can be of use to those attempting to collaborate with industry in a similar way. This guide is referenced by C.H.I.R.P. team members to guide similar concurrent and subsequent collaborations. While following these steps can be useful, these steps in isolation may not guarantee success. In situations where there is a lack of data availability, or the initial study was not productive, discontinuation of future collaborative projects should be considered if needed. If both parties were satisfied with the preliminary project, planning for the subsequent retrospective or prospective study should commence, beginning at step four if a new IRB protocol, application, or amendment is required. The planning in many cases occurs concurrently with the first study. C.H.I.R.P. has followed these steps with the goal of assessing consumer satisfaction and clinical outcomes of general users and the cardio-oncology patient population. This guide can help other research teams and students do the same in the field of cardio-oncology or other fields that stand to benefit from academic-industry collaboration based digital health research.

## Author contributions

JM and SAB: conception and design, data analysis, and interpretation. JM, MA, SP, RM, AS, AH, and SAB: drafting of the manuscript. JM, MA, SP, RM, AS, AH, RC, VZ, DA, BB, and SAB: critical revision. All authors: final approval of manuscript. All authors contributed to the article and approved the submitted version.

## Funding

This publication was supported by the National Center for Advancing Translational Sciences, National Institutes of Health, through Grant Nos. UL1TR001436 and KL2TR001438. Support was also provided by the Cancer Prevention Research Institute of Texas (RP180404), and this funding was not involved in the study design, collection, analysis, interpretation of data, the writing of this article, or the decision to submit it for publication.

## Conflict of interest

All of our authors are involved in digital health research, none of which inappropriately restricts or limits our analyses or publications. SAB serves as an advisor for HeartIn and NutriSense. SAB has not received compensation or research support for any work related to this project. The remaining authors declare that the research was conducted in the absence of any commercial or financial relationships that could be construed as a potential conflict of interest.

## Publisher's note

All claims expressed in this article are solely those of the authors and do not necessarily represent those of their affiliated organizations, or those of the publisher, the editors and the reviewers. Any product that may be evaluated in this article, or claim that may be made by its manufacturer, is not guaranteed or endorsed by the publisher.

## Author disclaimer

Its contents are solely the responsibility of the authors and do not necessarily represent the official views of the NIH.
